# Multiplex single-tier LymeSeek test improves sensitivity during the diagnostic gray zone of early Lyme disease

**DOI:** 10.1128/jcm.00879-26

**Published:** 2026-10-05

**Authors:** Holly Ahern, James Kirk, Phillip Stafford, Nikos Tsolakos, Mara Mandelia, Elizabeth J. Horn, Alison Rebman, John Aucott, Monica E. Embers

**Affiliations:** 1 State University of New York, Adirondack, Queensbury, New York, USA; 2 Aces Diagnostics Inc., Paradise Valley, Arizona, USA; 3 Arizona State University, School of Life Sciences 7864, Tempe, Arizona, USA; 4 Protavio Ltd. 674176 , Athens, Greece; 5 Lyme Disease Biobank, Portland, Oregon, USA; 6 Division of Rheumatology, Department of Medicine, Lyme Disease Research Center, Johns Hopkins University School of Medicine 1500, Baltimore, Maryland, USA; 7 Division of Immunology, Tulane National Biomedical Research Center, Tulane University Health Sciences 101404 , Covington, Louisiana, USA; Mayo Clinic Minnesota, Rochester, Minnesota, USA

**Keywords:** machine learning, Luminex, serology, single-tier, diagnostics, Lyme disease

## Abstract

Lyme disease, caused by the spirochete *Borrelia burgdorferi*, is the most common vector-borne disease in the U.S. and a growing public health concern due to the clinically challenging nature of the disease. Prompt antibiotic treatment early in the infection results in the best patient outcomes, while delayed treatment increases the risk of developing debilitating chronic symptoms. Because standard-of-care diagnostic tests (standardized and modified two-tier tests) have low sensitivity during the first few weeks of infection, current recommendations for diagnosing early Lyme disease rely heavily on observation and recognition of erythema migrans (EM). However, a significant proportion of Lyme disease patients develop an atypical-appearing rash, or no rash is observed. To improve diagnostic accuracy, we developed LymeSeek, a microbead-based multiplex flow immunoassay performed as a single test that detects binding of IgM and IgG antibodies to a novel combination of eight *B. burgdorferi* antigens. Fluorescent signals from bound antibodies are translated into a disease probability and a single test result—either detected or not detected. Overall test sensitivity across all disease stages was over 90%, with no loss of specificity. The LymeSeek test performed well in blinded panels, correctly differentiating serologically positive cases from both disease and non-disease controls. The test also identified early Lyme disease cases, with and without an observed EM rash, that were negative on the current standard-of-care two-tier serological tests. The LymeSeek test has the potential to increase diagnostic accuracy and improve patient outcomes, particularly during the diagnostic gray zone of early Lyme disease.

**IMPORTANCE:**

Standard and modified two-tier serological tests for Lyme disease are not sensitive enough to diagnose patients within the first few weeks of infection, when antibiotic treatment is recognized as being most effective. This report describes a single-tier multiplex immunoassay that utilizes detection of 16 diagnostic antibodies against eight *Borrelia* antigens and interpretation by a machine learning-developed algorithm that demonstrates sensitivity high enough to accurately diagnose over 90% of Lyme disease cases, including those in the early diagnostic gray zone period when diagnostic uncertainty is highest. Specificity is high enough to eliminate the need for a second-tier test.

## INTRODUCTION

Lyme disease (LD) is a bacterial infection caused by spirochetes of the *Borrelia burgdorferi sensu lato* complex, which includes several genospecies. LD is the most common tick-borne and vector-borne disease in the United States and Europe. Lyme disease symptoms are recognized as occurring in stages, with symptoms varying in severity and presentation depending on pathogen and host factors. Early LD diagnosis is based mostly on clinical signs, specifically a known tick bite followed by an erythema migrans (EM) rash, which is an established clinical marker of the disease. However, a significant portion of LD patients do not present with a classic EM rash, or a rash is not observed (1, 2). For such early cases, two-tier serologic tests are recommended as an aid to diagnosis, but the limitations of both standard two-tier testing (STTT) and newer modified two-tier tests (MTTT) during a serological gray zone of 1–3 weeks are well recognized, with sensitivity below 50% (3). Variable EM presentations and poor test sensitivity impede clinical diagnosis at this early stage, when antibiotic treatment shows the greatest efficacy.

The majority of patients who are diagnosed and treated during the EM rash stage of LD recover fully from the illness within 6 months (4). Late or missed LD diagnosis can result in dissemination of the bacteria to the heart, joints, and nervous system tissues, causing cardiac, arthritic, and neurological symptoms that can be debilitating (5). Approximately 10%–20% of ideally diagnosed and treated LD patients develop persisting symptoms, which is referred to as post-treatment Lyme disease (PTLD) (6, 7). Progression to Lyme infection-associated chronic illness (Lyme-IACI), for which the cause and treatment have not been established, is much higher in patients with delayed diagnosis (8). Lyme-IACI is associated with significant morbidity, including profound fatigue, muscle and joint pain, and neurological and neuropsychiatric complaints that can persist for several years (9, 10). Between the population of patients who develop PTLD following early diagnosis and those with delayed or initial misdiagnosis, the rate of chronic illness may be as high as 50% (11).

Our goal was to improve LD patient outcomes by developing a single-tier diagnostic test with high sensitivity and specificity during the early infection period, thereby overcoming the current diagnostic limitations and enabling earlier antibiotic treatment.

The test that we developed is a multiplex bead-based flow immunoassay that detects IgM and IgG antibody binding to a novel combination of eight *B. burgdorferi* antigens, expressed across all stages of infection. Antibody binding is detected fluorescently using standard clinical laboratory instrumentation, and fluorescent output is analyzed using a proprietary advanced machine learning-developed binomial classification algorithm that captures the heterogeneity of the immune response across Lyme disease stages and presentations. We validated test performance using standard diagnostic performance metrics. The performance of the immunoassay and software combination (LymeSeek) was compared against both STTT and MTTT first-tier ELISA assays using panels of well-characterized Lyme disease patient and control sera.

## MATERIALS AND METHODS

### Serum samples

For algorithm training and testing, we assembled a large set of well-characterized Lyme disease patients and endemic, non-endemic, and look-alike disease control serum samples. These were obtained from the CDC Lyme Serum Repository (Fort Collins, CO, USA) (12), the Lyme Disease Biobank (Portland, OR, USA) (13), and the Johns Hopkins University “Study of Lyme disease Immunology and Clinical Events” (SLICE) study (Baltimore, MD, USA) (14). Non-endemic control samples were also obtained from Sonora Quest Laboratories in Phoenix, AZ, USA. Additional samples were obtained from two commercial vendors: Complex Antibodies Inc. in Florida and Central BioHub in Germany. All samples were maintained and shipped to Kryosphere (Raleigh, NC, USA) for clinical testing.

### Immunoassay principles

**Fig 1:**
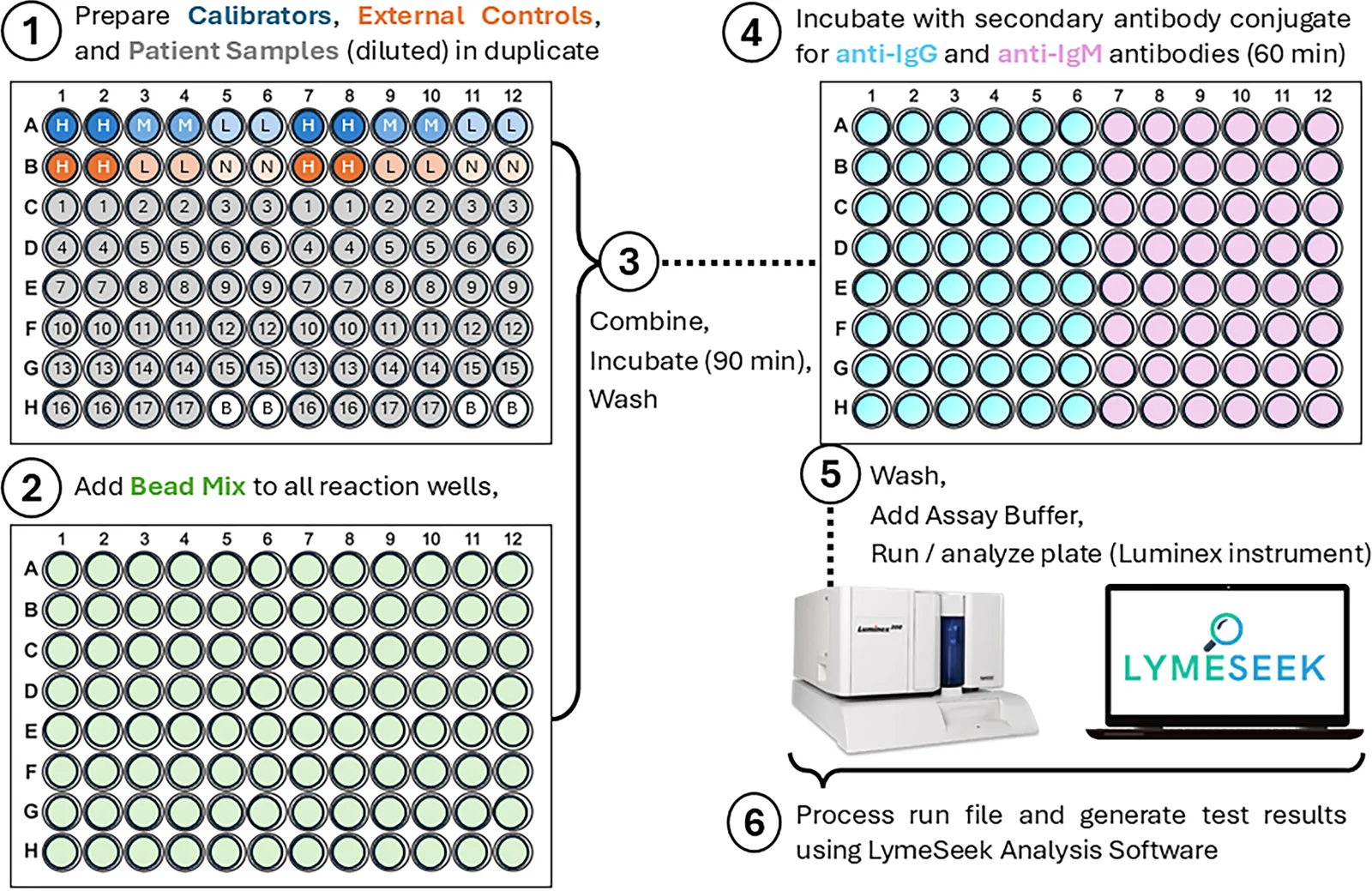
Workflow of the LymeSeek test, a bead-based flow immunoassay interpreted by a proprietary algorithm designed for clinical laboratory implementation.

The LymeSeek test is a bead-based flow immunoassay performed in a 96-well standard flat-bottom microtiter plate format utilizing Luminex xMAP technology to detect IgM and IgG antibody binding to antigens conjugated to color-coded beads (Fig. 1). Of the 10 antigens initially included during assay development, the final antigen panel consists of eight *Borrelia* antigens (OspC, DbpA, OppA2, ErpQ, OspE, P35, P66, and one synthetic peptide [C6]). The two antigens that were removed (OspA and OspC1 peptide) did not change the sensitivity or specificity of the assay based on computational analysis. The LymeSeek software includes a proprietary machine learning-developed algorithm trained on case and control samples. Test design, methodology (15), and algorithm development have been previously described (16). Briefly, purified proteins and the C6 peptide were coupled to Luminex beads using the Bio-Plex Amine Coupling kit (Bio-Rad). A 1:200 dilution of serum is the standard amount added to the bead mix for binding reactions. Both IgG and IgM are detected with anti-human Ig-phycoerythrin-coupled secondary antibodies (Southern Biotech).

The test also includes a set of novel calibrators developed by Protavio Ltd. (Athens, Greece), consisting of a mix of chimeric murine/human monoclonal antibodies against all antigens included in the bead mix. For test calibration, the chimeric monoclonal antibodies showed equivalent performance to human positive control patient serum samples, ensuring a continuous supply of antibodies for LymeSeek test kit production.

### LymeSeek algorithm development

**Fig 2:**
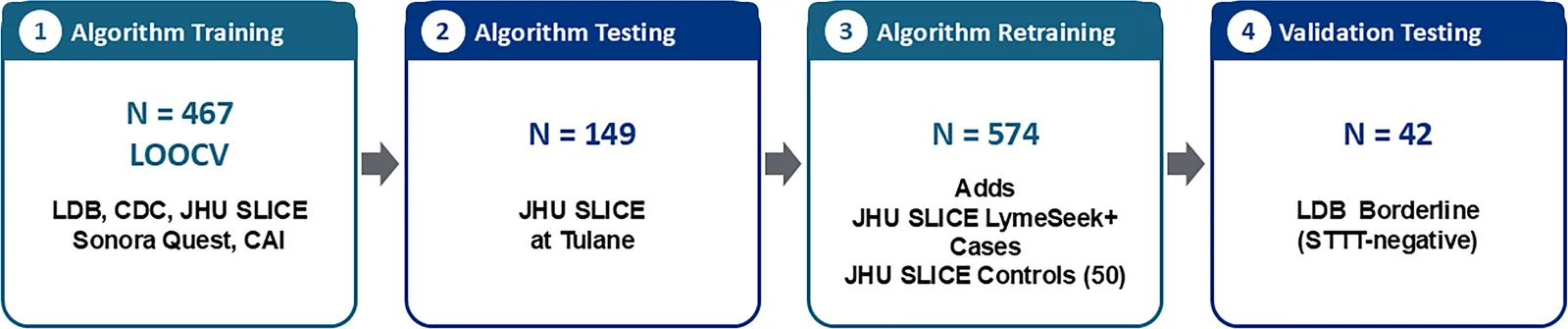
LymeSeek algorithm development established the baseline algorithm using 467 well-characterized case and control samples for training (panel 1) and 149 samples for testing (panel 2). The algorithm was retrained on 574 additional cases and controls (panel 3) and validated against clinically diagnosed, STTT-negative cases (panel 4).

Development and initial validation of the proprietary diagnostic algorithm with a boosted decision tree machine learning model have been previously described (16). The LymeSeek algorithm was developed with a stepwise workflow training using well-characterized Lyme disease case and control samples, followed by testing, retraining, and validation testing (Fig. 2). The final result is either above the cutoff value for weighted antigen responses (detected) or below the cutoff value (not detected).

The initial LymeSeek training/testing cohort included 467 serum samples obtained from highly respected Lyme disease research biorepositories (Lyme Disease Biobank, Johns Hopkins University SLICE study, and the CDC Lyme Serum Repository) and Complex Antibodies Inc., a commercial vendor of remnant biospecimens collected under IRB approval protocols (Table 1). The Johns Hopkins SLICE study enrolls patients with early Lyme disease to research immune responses and symptom progression over time. Participants are followed from the time of first diagnosis with early Lyme disease through 12 months of post-treatment convalescence. Blood samples obtained at all time points were tested by STTT at Quest Laboratories. LDB enrolls participants with signs and symptoms of early Lyme disease with and without EM. All samples, including controls, are tested at the end of each season using first- and second-tier assays, and STTT algorithms are retrospectively applied (13, 17).

**TABLE 1:** Sources and characteristics of serum samples used for development and validation of LymeSeek

Source	Cohort total (*N*)	Lyme disease positive				Lyme disease negative		
		Early	Convalescent	Late	Other	Healthy non-endemic	Healthy endemic	Lookalike disease
Lyme Disease Biobank	178	43	22	–^*c*^	–	–	113	–
CDC Research Panel I	10	4	3	1	2 neuro	–	–	–
CDC Research Panel II	87	10	9	4	3 neuro, 1 cardiac	12	12	36
Johns Hopkins University SLICE (Longitudinal)	58	30^*a*^	28^*b*^	–	–	–	–	–
Sonora Quest (AZ, USA)	94	–	–	–	–	94	–	–
Complex Antibodies Inc.	40	–	–	–	–	–	–	40
Total	467	87	62	5	6	106	125	76

^*a*^ The JHU SLICE Longitudinal sera set included Visit 1 (0–72 h after illness onset/treatment) samples.

^*b*^ The JHU SLICE Longitudinal sera set included Visit 3 (2 months post-diagnosis/treatment) samples.

^*c*^ –, category not included.

To evaluate whether LymeSeek could be implemented more broadly to detect Lyme borreliosis, 49 samples from patients diagnosed with *Borrelia afzelii*, the most common cause of Lyme borreliosis in the European Union (EU), and controls (*n* = 49) were obtained from Central BioHub in Germany and tested using the LymeSeek test.

Reproducibility was evaluated during technology transfer from Tulane University, where the original immunoassay was developed and verified, to the manufacturing environment at Protavio Ltd., a contract development and manufacturing organization in Athens, Greece. The proteins and peptides used in the assay were reconstructed from DNA sequences at Protavio Ltd. with no additional access to training data, classifier, or predictions used during the Tulane phase of development. This transition deliberately introduced multiple uncontrolled variables, including different reagent lots, instruments, operators, peptide synthesis batches, protein expression systems, and sample populations. The algorithm was intentionally not recalibrated to better evaluate the impact of this transition.

## RESULTS

### Overall test performance

Training of the initial LymeSeek algorithm on 467 samples included 160 well-qualified Lyme disease cases enrolled across a time frame starting within a few days of onset (presentation of the EM rash) through disseminated and late-stage Lyme disease. A total of 231 control samples from both endemic and non-endemic regions in the U.S. and 76 control samples from look-alike diseases were included.

Across all timepoints, the LymeSeek algorithm achieved a sensitivity of 90%, without loss of specificity based on Leave-One-Out Cross-Validation (Table 2).

**TABLE 2:** Performance of the LymeSeek test on 467 samples during algorithm development^*a*^

	LymeSeek	
	Positive	Negative
True positive	144	16
True negative	23	284
AUC (ROC)	0.97	
Sensitivity	0.90	
Specificity	0.93	
Accuracy	0.92	

^*a*^ Testing was performed using a prototype LymeSeek test kit and completed at Tulane University.

### Evaluation of the Johns Hopkins University SLICE study panels

**Fig 3:**
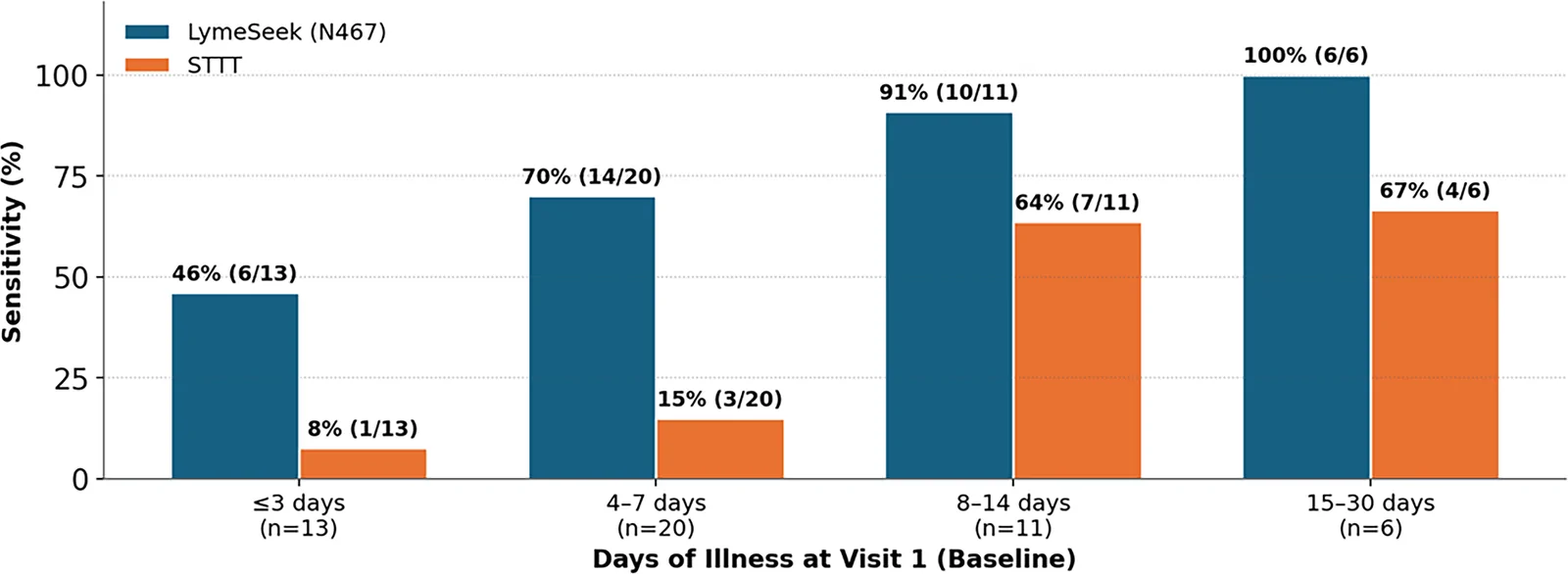
Direct comparison of test results returned by the LymeSeek test vs STTT within 1 month of disease onset, according to days of illness in 50 early Lyme disease cases from JHU SLICE.

Using the locked algorithm from the training set, a broader set was tested for assay performance. Samples from 50 early Lyme disease patients from the JHU SLICE Visit 1 cohort were then tested using the LymeSeek immunoassay/locked algorithm. The LymeSeek test correctly identified 100% of the early Lyme disease cases 15–30 days after illness onset, defined as presentation of the EM rash (Fig. 3). The test significantly outperformed STTT at the earliest time point (within 3 days of onset) and detected over 90% of Lyme disease cases within 2 weeks.

### Test performance in the early Lyme disease diagnostic gray zone

To enhance machine learning and improve test performance during the early window, we added additional well-characterized, clinically diagnosed but two-tier testing negative cases and retrained the algorithm on 574 samples. The new algorithm was used to evaluate the performance of the LymeSeek test in a cohort of 30 cases from the Lyme Disease Biobank that were STTT negative. This cohort included samples from participants enrolled with signs and symptoms of early Lyme disease with and without suspected EM (13, 17). These cases are highly representative of a gray zone where Lyme disease diagnosis cannot be confirmed by the current standard-of-care STTT or MTTT approach.

In early Lyme disease cases that were serologically negative by two-tier testing, LymeSeek detected 75% of the cases enrolled with an observed EM, and 60% of the cases enrolled with constitutional symptoms but without EM (Table 3).

**TABLE 3:** Summary of 30 early samples from the Lyme Disease Biobank tested with LymeSeek that were clinically diagnosed with Lyme disease at the time of enrollment but were STTT negative

LDB clinical group	*N*	Observed EM rash	STTT positive	LymeSeek positive
Enrolled with EM > 5 cm	16	16/16 (100%)	0/16 (0%)	12/16 (75%)
Enrolled with EM < 5 cm	4	4/4 (100%)	0/4 (0%)	3/4 (75%)
Enrolled without EM	10	0/10 (0%)	0/10 (0%)	6/10 (60%)
Total	30	20/30 (67%)	0/30 (0%)	21/30 (70%)

**Fig 4:**
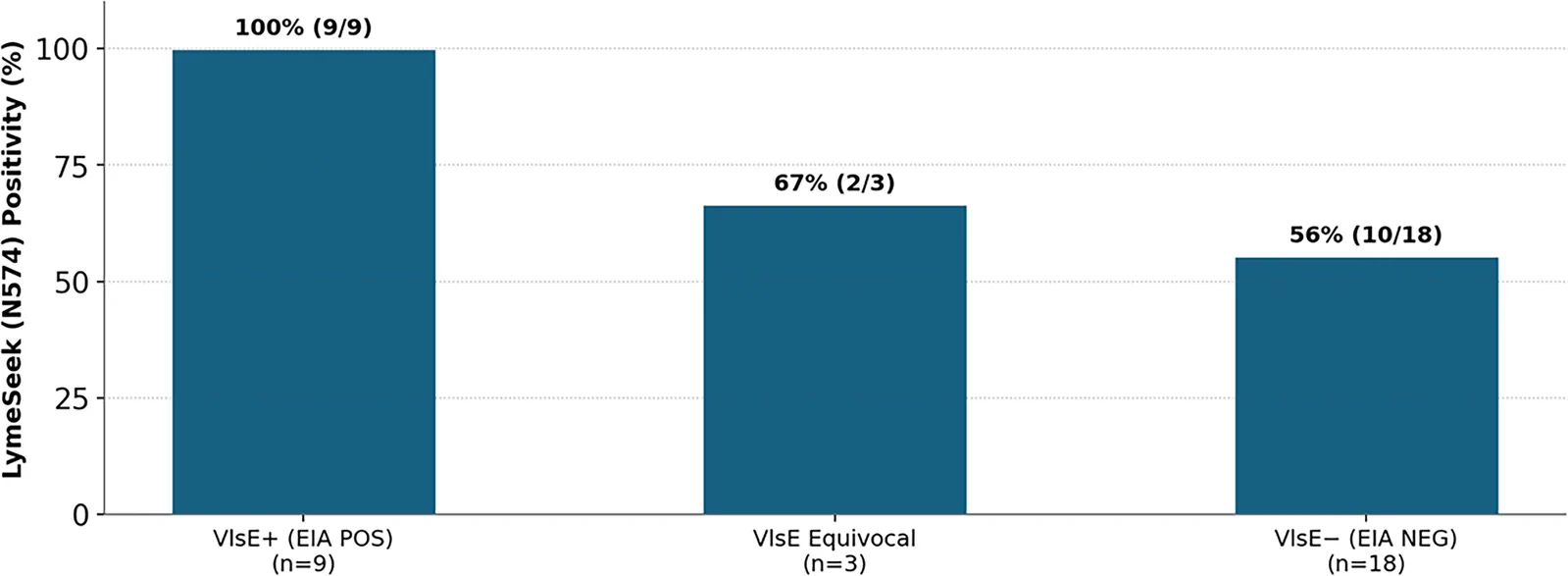
LymeSeek positivity compared to testing by VlsE/pepC10 EIA in 39 early Lyme samples from LDB.

In the LDB cohort, all samples that were positive by VlsE/pepC10 EIA, the first-tier test used in STTT or MTTT, were also LymeSeek positive. The LymeSeek test detected 56% of VlsE/pepC10 EIA-negative cases, which are representative of confirmed Lyme disease cases that would not be detected using the current standard-of-care two-tier tests, and diagnosis of Lyme disease using the STTT or MTTT approach would be missed (Fig. 4).

**Fig 5:**
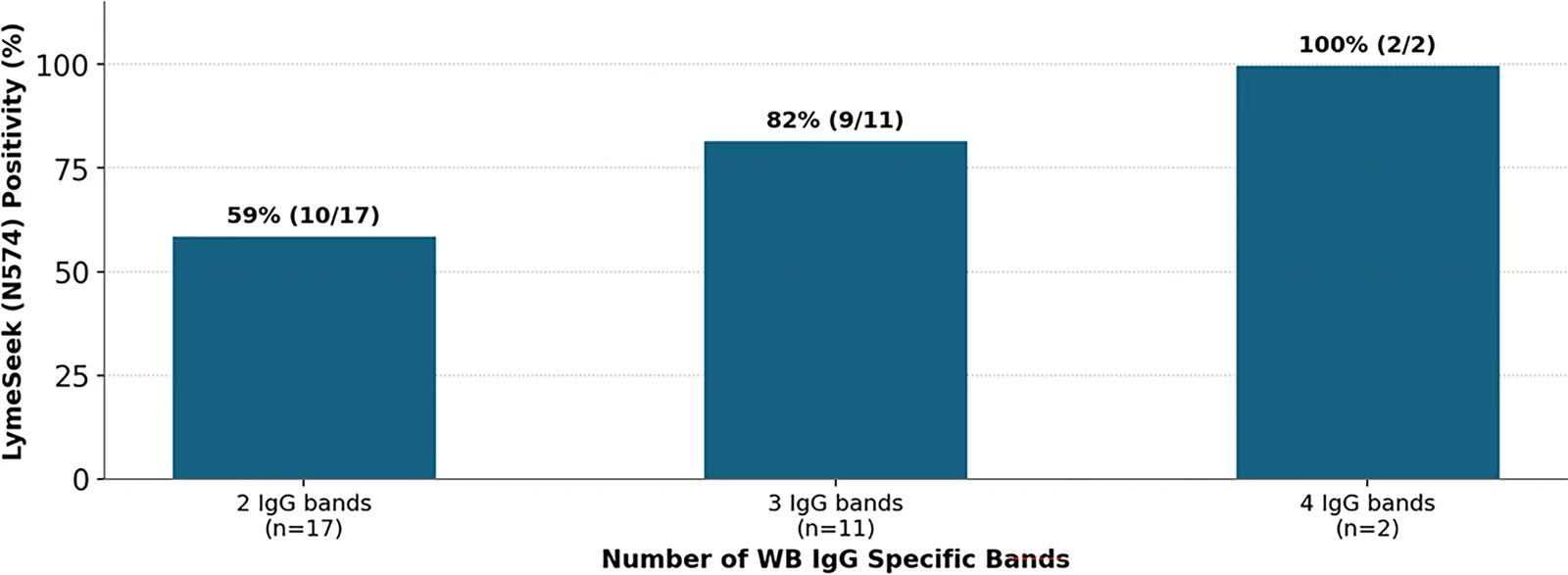
LymeSeek positivity compared to the number of IgG bands on the Western blot in 30 early Lyme samples from LDB.

To further confirm that a positive LymeSeek test was a true indicator of *Borrelia* infection, we compared the test with the number of positive bands observed on an IgG Western blot. LymeSeek positivity rate increased with increasing number of IgG bands observed on the Western blot (59% with two bands, 82% with three bands, and 100% with four bands), indicating LymeSeek test results are consistent with the cumulative serological evidence obtained by STTT (Fig. 5).

### Test accuracy for Lyme borreliosis common in EU

*Borrelia burgdorferi* is the predominant genospecies identified in ticks and in patients with Lyme disease in the U.S. and Canada. In European countries, Lyme borreliosis is also attributed to infection with *B. afzelii* and *B. garinii*. To evaluate whether the LymeSeek test would be able to detect Lyme borreliosis caused by genospecies of *Borrelia* other than *B. burgdorferi*, we obtained 21 serum samples from Lyme borreliosis cases caused by *B. afzelii* and 28 controls from Central BioHub GmbH, a commercial vendor in Germany.

After testing, the LymeSeek test identified 17/21 (81%) of Lyme borreliosis cases and only 4/28 (14%) of the controls, while specificity remained above 85% in this cohort (Table 4). Case information obtained from the vendor included only that the samples were Lyme borreliosis with test results for *B. afzelii*; therefore, it cannot be ruled out that the false positive control cases may have been infected with a different *Borrelia* strain, since *B. burgdorferi sensu lato* strains are also found across Europe.

**TABLE 4:** Confusion matrix to evaluate LymeSeek performance with samples of Lyme borreliosis with positive serology results for *B. afzelii*

	LymeSeek positive	LymeSeek negative
Lyme borreliosis	17	4
Negative controls	4	24

### Assessment of test reproducibility

In a preliminary evaluation of reproducibility, the LymeSeek assay developed at Tulane University was rebuilt and performed on the manufacturer’s instrumentation. A distinct 259-person cohort was evaluated using the LymeSeek immunoassay rebuilt starting from antigen DNA sequences at Protavio, Ltd. The LymeSeek immunoassay/locked classifier achieved an AUC of 0.98 (0.96–1.00), sensitivity 0.90 (0.78–0.97), specificity 0.98 (0.95–0.99), positive likelihood ratio 45, and negative likelihood ratio 0.10, which matched the internal validation, confirming that the LymeSeek test maintained diagnostic accuracy after transition from an academic research lab to the manufacturing environment. In practice, the test report will include MFI values, cutoff value, and test result reported as detected or not detected based on whether it is above or below the threshold developed by the algorithm.

## DISCUSSION

As an aid to diagnosis of Lyme disease, the LymeSeek test provides one single, actionable test result based on a pattern of antibody binding. The algorithm interrogates this reactivity to reconstruct patient-specific immune responses. This addresses a fundamental challenge noted with the current serological tests for Lyme disease; patients infected with *Borrelia* mount variable immune responses (18, 19) that single thresholds applied to individual antigens in the current MTTT and STTT diagnostic tests do not entirely capture. From a clinical laboratory perspective, MTTT is considered an improvement over STTT because the tests are easier to perform; however, overall test sensitivity is only slightly improved.

While a negative interpretation of STTT or MTTT does not actually rule out a diagnosis of Lyme disease, current medical guidelines assert that antibiotic treatment is not indicated if the test result is negative (20). Diagnostic uncertainty due to the low sensitivity of current diagnostic tests therefore impacts patient care. A survey of clinicians who see patients with Lyme-IACI after antibiotic treatment reported that false-negative lab tests during early infection were a major roadblock to early diagnosis and treatment (21).

We obtained samples from the LDB, JHU SLICE, and CDC Research Panel 1 that were highly characterized for Lyme disease status in terms of clinical presentation and other criteria. These samples included Lyme disease cases, with and without an EM rash, that were negative for two-tier serology. This represents the diagnostic gray zone for early Lyme disease, where diagnosis cannot be confirmed by laboratory tests and patients may not receive antibiotic treatment.

The LymeSeek test showed significantly improved sensitivity over STTT and MTTT across all stages, but particularly during early infection, highlighting its potential to improve diagnosis and reduce the rate of chronic illness among Lyme disease patients. The test’s high negative predictive value (0.95, 95% CI: 0.91–0.97) would also potentially reduce unnecessary antibiotic prescriptions while ensuring treatment for true cases, particularly in non-endemic regions where provider education on Lyme disease risk lags. We surmise that the increased sensitivity is due to a combination of the increased analytical sensitivity of antibody detection on the Luminex platform, the selection of antigens, and the inclusion of both isotypes for specific reactivity to those antigens. Compared to the STTT, the threshold for positive detection of antibody responses is reduced, enhancing sensitivity without decreasing specificity.

To assess test reproducibility, the LymeSeek assay developed at Tulane University was rebuilt and tested at Protavio, Ltd. Raw fluorescence data were returned with anonymized identifiers. The LymeSeek test performance metrics remained consistent when performed in these very different testing environments. This was a preliminary evaluation only, and additional reproducibility studies will be completed during FDA clinical study proceedings.

This study has limitations. The serum samples used in this study were characterized as Lyme disease cases on the basis of diagnostic criteria (skin lesion and two-tier serological testing) with a history of poor clinical utility. In previous and recent epidemiological and medical studies of Lyme disease, observation of the EM skin lesion heavily influences identification of Lyme disease cases; however, the rate of Lyme disease in *Borrelia*-infected patients infected by tick bite who do not develop an EM (2), or by perinatal transmission of *Borrelia* during pregnancy (22), is unknown. Similarly, not all skin lesions suspected to be EM are caused by *Borrelia* infection (2). The limitations of current two-tier serological tests are already well established (3).

As is true for other serological tests for Lyme disease, a positive LymeSeek test may not be able to distinguish active *Borrelia* infection from persistent antibodies from a previous infection. Testing of additional samples from diagnosed and treated Lyme disease patients who returned to health is underway to evaluate whether the algorithm can distinguish antibody responses in early infection from those in resolved infection.

In conclusion, the LymeSeek test is a promising single-tier replacement for the current standard-of-care two-tier test, providing higher sensitivity without loss of specificity in comparison with both STTT and MTTT. The higher sensitivity of LymeSeek in the early diagnostic gray zone window of *Borrelia* infection will better inform treatment decisions during the critical period where antibiotic treatment reduces the risk of Lyme-IACI. The test was designed to be performed as a single-tier test in clinical laboratories using a format similar to other diagnostic immunoassays, with a goal of improving patient access to an accurate diagnostic test for Lyme disease following a traditional route for diagnostic testing of infectious diseases. FDA clearance of LymeSeek as an *in vitro* diagnostic test following a 510(k) regulatory pathway has been initiated.

## Data Availability

The de-identified data that support the findings of this study are not publicly available because they contain information derived from patient records and are subject to privacy protections. Qualified researchers may request access to the data from the corresponding author. Requests will be reviewed in accordance with institutional policies, ethical requirements, and applicable data-sharing agreements.

## SUPPLEMENTAL MATERIAL

Fig. S1 and S2 jcm.00879-26-s0001.pdf Linearity plots.

ASM does not own the copyrights to Supplemental Material that may be linked to, or accessed through, an article. The authors have granted ASM a non-exclusive, world-wide license to publish the Supplemental Material files. Please contact the corresponding author directly for reuse.
