# Supplementary material for: Multiplex single-tier LymeSeek test improves sensitivity during the diagnostic gray zone of early Lyme disease
Source: J Clin Microbiol. 2026 Oct 5;64(11):e00879-26. doi: 10.1128/jcm.00879-26 (PMC13753739; doi:10.1128/jcm.00879-26)

Supplemental Figure 1. Linear fits across all analytes in the IgG assay, along with the corresponding equations and R2 values. Dotted lines indicate the reference line (y = x), representing perfect agreement between expected and measured concentrations.

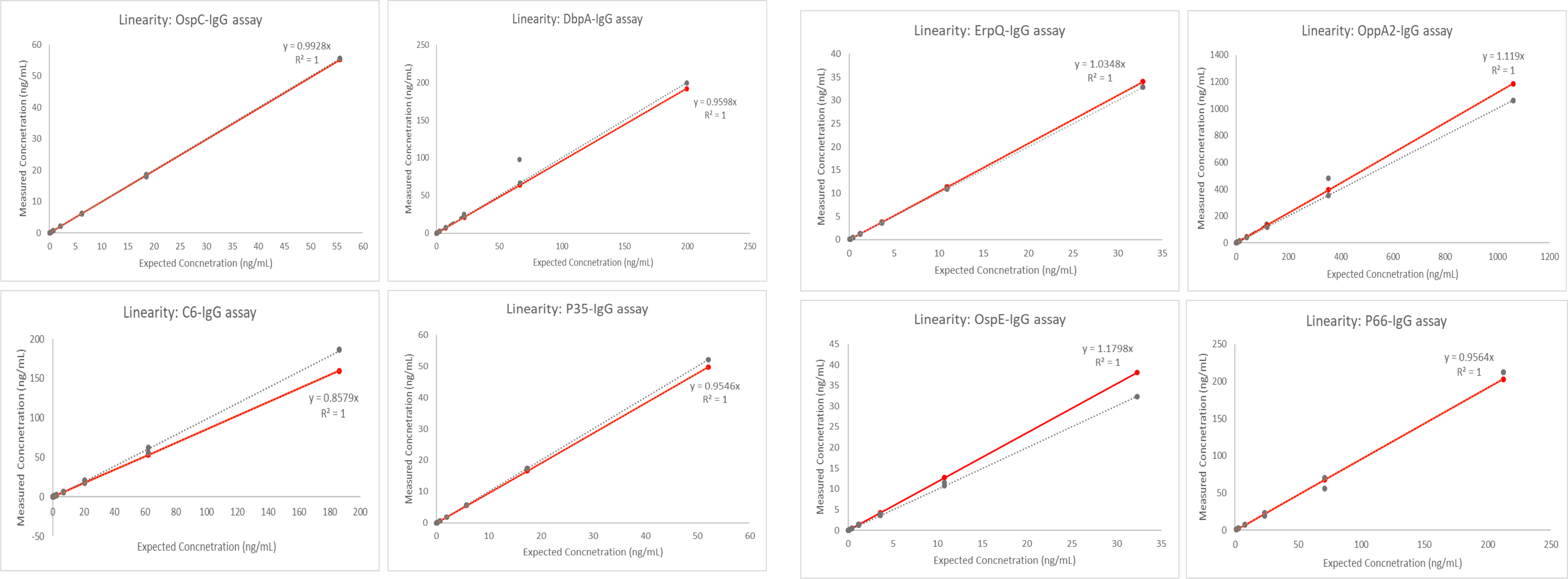

Supplemental Figure 2. Linear fits across all analytes in the IgM assay, along with the corresponding equations and R2 values. Dotted lines indicate the reference line (y = x), representing perfect agreement between expected and measured concentrations.

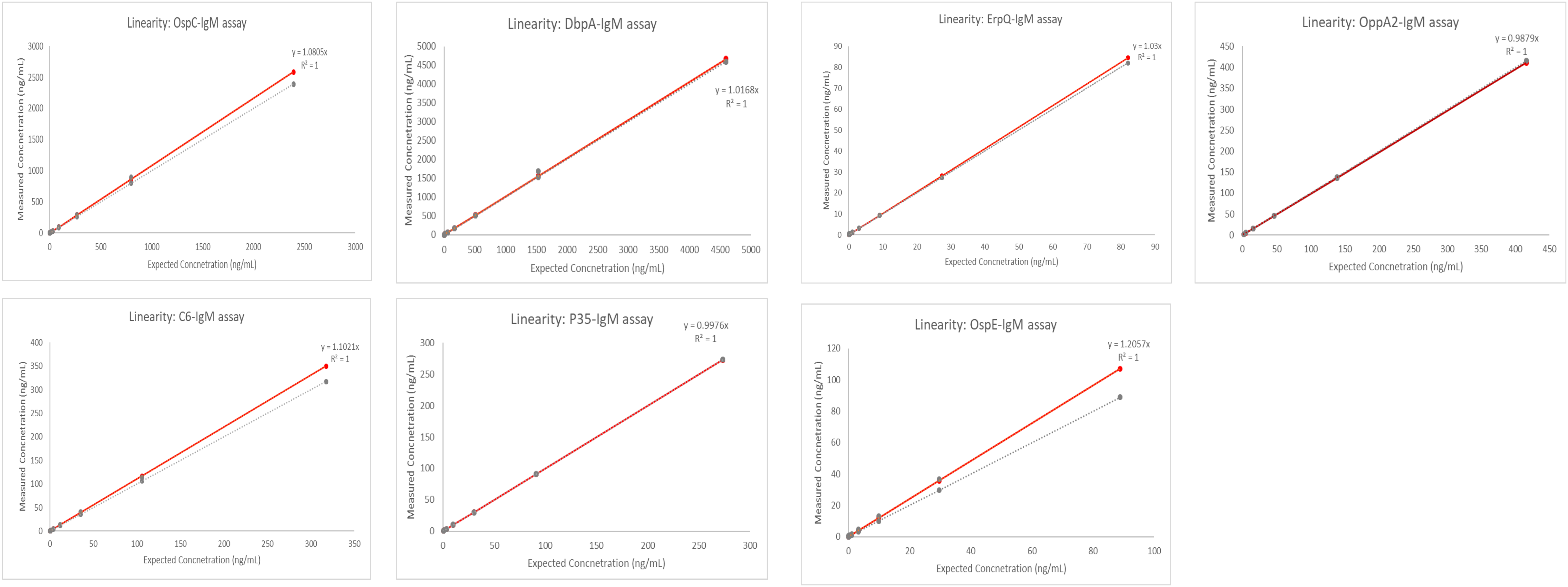

Supplement: Supplementary file 1 [file jcm.00879-26-s0001.pdf]
